# Compound Library Screening Identified Cardiac Glycoside Digitoxin as an Effective Growth Inhibitor of Gefitinib-Resistant Non-Small Cell Lung Cancer via Downregulation of α-Tubulin and Inhibition of Microtubule Formation

**DOI:** 10.3390/molecules21030374

**Published:** 2016-03-18

**Authors:** Yi-Ze Zhang, Xi Chen, Xing-Xing Fan, Jian-Xing He, Jun Huang, Da-Kai Xiao, Yan-Ling Zhou, Sen-You Zheng, Jia-Hui Xu, Xiao-Jun Yao, Liang Liu, Elaine Lai-Han Leung

**Affiliations:** 1 State Key Laboratory of Quality Research in Chinese Medicine/Macau Institute for Applied Research in Medicine and Health, Macau University of Science and Technology, Taipa, Macau SAR, China; mmmiazh@gmail.com (Y.-Z.Z.); aurora329@163.com (X.C.); fxx022@163.com (X.-X.F.); ylzhou@must.edu.mo (Y.-L.Z.); steven4238@hotmail.com (S.-Y.Z.); ntxjhui@gmail.com (J.-H.X.); xjyao@must.edu.mo (X.-J.Y.); 2 Department of Thoracic Surgery, The 1st Affiliated Hospital of Guangzhou Medical University, Guangzhou Institute of Respiratory Disease, State Key Laboratory of Respiratory Disease, Guangzhou 510120, China; hejx@vip.163.com (J.-X.H.); kingmal17@126.com (J.H.); oneplusten@126.com (D.-K.X.)

**Keywords:** natural product compound library, Digitoxin, NSCLC, EGFR, TKI resistance, microtubule, cell cycle, α-tubulin

## Abstract

Non-small-cell lung cancer (NSCLC) dominates over 85% of all lung cancer cases. Epidermal growth factor receptor (EGFR) activating mutation is a common situation in NSCLC. In the clinic, molecular-targeting with Gefitinib as a tyrosine kinase inhibitor (TKI) for EGFR downstream signaling is initially effective. However, drug resistance frequently happens due to additional mutation on EGFR, such as substitution from threonine to methionine at amino acid position 790 (T790M). In this study, we screened a traditional Chinese medicine (TCM) compound library consisting of 800 single compounds in TKI-resistance NSCLC H1975 cells, which contains substitutions from leucine to arginine at amino acid 858 (L858R) and T790M mutation on EGFR. Attractively, among these compounds there are 24 compounds CC_50_ of which was less than 2.5 μM were identified. We have further investigated the mechanism of the most effective one, Digitoxin. It showed a significantly cytotoxic effect in H1975 cells by causing G2 phase arrest, also remarkably activated 5′ adenosine monophosphate-activated protein kinase (AMPK). Moreover, we first proved that Digitoxin suppressed microtubule formation through decreasing α-tubulin. Therefore, it confirmed that Digitoxin effectively depressed the growth of TKI-resistance NSCLC H1975 cells by inhibiting microtubule polymerization and inducing cell cycle arrest.

## 1. Introduction

According to the World Health Organization (WHO), lung cancer is becoming the major reason of cancer-caused death, and accounts for 20% of all cancer death cases and causes millions of deaths per year [1]. Lung cancer incidence has still been increasing which leads to a big social burden [2,3]. There are two types of lung cancer, non-small-cell lung cancer (NSCLC) and small cell lung cancer (SCLC), while NSCLC is the most common histological types and dominates 85% of all lung cancer cases [4].

Nowadays, conventionally treatments are used for NSCLC including surgical operation, radiotherapy and chemotherapy [5,6,7]. In addition, some new treatments are applied to against NSCLC especially molecular-targeted therapy. For target-based therapy, the application of tyrosine kinase inhibitor (TKI) has been well testified to benefit NSCLC patients with epidermal growth factor receptor (EGFR) mutations. It is highly expressed on the cell surface of more than 60% NSCLC cells [8]. Therefore, it is increasingly wide utilization in clinic as molecular targets as individual comprehensive therapy for NSCLC patients with EGFR mutation.

Gefitinib, first generation of TKI, is clinically-proved effective for patients who harbor an activating substitution from leucine to arginine at amino acid 858 (L858R) point mutation and in-frame exon 19 deletion on EGFR, which are biomarkers for good response to Gefitinib [9,10]. Initially, it was an outstanding therapy for most EGFR mutation harboring patients; however, after around one year, the major additional EGFR mutation such as substitution from threonine to methionine at amino acid position 790 (T790M) occurred which led to eventual TKI resistant [11].

In order to overcome the first-generation TKI resistance, pharmaceutical companies spent extensive efforts in developing second and third generation TKI, such as Afatinib which is currently FDA-approved [12]; however, eventually, like Gefitinib, Afatinib also comes with resistance reports [13] and it showed more adverse effects when compared with Gefitinib [13,14]. All these clinical facts indicate that more potential drug candidates should be explored.

In recent years, anti-cancer small molecules have been identified from natural products. Most of these compounds were found from Traditional Chinese Medicine (TCM) herbs with exquisite activity against cancer. For example, Camptothecin (from *Camptotheca acuminata*) [15,16], Homoharringtonine (from *Cephalotaxus harringtonia*) manifested anti-cancer function [17,18]. Paclitaxel was originally isolated from the bark of the yew tree *Taxus brevifolia* showed strong anti-cancer capability [19,20]. Willow bark extract could induce apoptosis and showed anti-proliferation activity in lung cancer [21]. Curcumin, which is a compound isolated from turmeric, targets cancer survival pathways and also prevents drug resistance [22].

Our preliminary work indicated that Celastrol, an isolated single compound from Chinese herb, caused apoptotic effect on Gefitinib-resistant NSCLC cell lines H1975 and H1650 [23]. Therefore, in this study, we aim to high-throughput screen a compound library composed of 800 single compounds purified from natural products to further identify effective compound on H1975. H1975 cell line with EGFR^T790M/L858R^ double mutation that resists to Gefitinib and control A549 cell line with wild-type (WT) EGFR were taken as objective for compound testing.

## 2. Results

### 2.1. Twenty-Four Compounds Were Shortlisted from a Natural Product Library Consisting of Compounds by Comparing Their Cytotoxicity in Human NSCLC H1975 and A549 Cells

**Figure 1:**
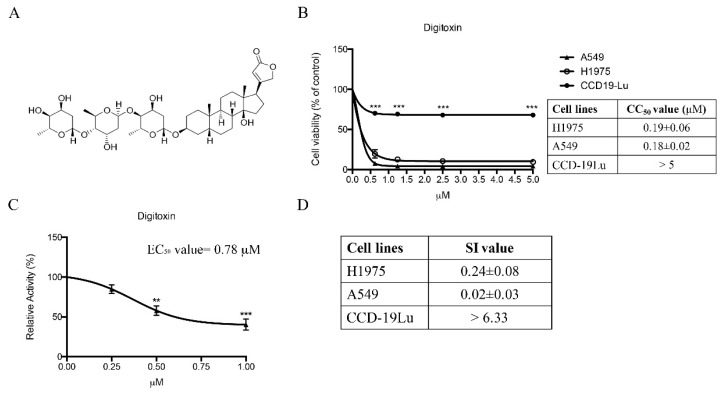
Cytotoxicy of Digitoxin. (**A**) Chemical structure of Digitoxin; (**B**) MTT assay results of Digitoxin on H1975 cells, A549 cells, and CCD-19Lu cells after 72 h treatment, respectively; (**C**) *In vitro* enzymatic assay of Na^+^/K^+^-ATPase; (**D**) SI values of H1975 cells, A549 cells, and CCD-19Lu cells respectively. All data were presented as mean ± SEM (*n* = 4, ** *p* < 0.01, *** *p* < 0.001) *vs.* vehicle control.

3-(4,5-Dimethylthiazol-2-yl)-2,5-diphenyl tetrazolium bromide (MTT) assay was used to detect cell inhibition rate of 800 candidate compounds on H1975 cells and A549 cells which harbors EGFR wild type (WT). All 800 compounds were tested in both cell lines for 72 h as preliminary screening at the concentration range of 0, 2.5, 5 and 10 μM and only 24 compounds showed CC_50_ values less than 2.5 μM in both cell lines, which were shortlisted in ascending order in Table 1. As shown in Table 1, Digitoxin has the highest cytotoxicity in H1975 cells, whose CC_50_ value was 0.19 ± 0.06 μM. These data implied that low dose of Digitoxin strongly effected on cells regardless of EGFR type, suggesting although Digitoxin had no selectivity for EGFR wild type and mutated NSCLC cells, is still useful in killing Gefitinib-resistance NSCLC cells. We further determined the cytotoxic effect of Digitoxin on normal lung fibroblast CCD-19Lu cells. Surprisingly, we found that the CC_50_ value of Digitoxin in H1975 cells was more than 25-fold lower than that of CCD-19Lu cells, which suggested that Digitoxin has strong inhibition selectivity in NSCLC cells (Figure 1B). In our result (Figure 1C), the EC_50_ value of Digitoxin was 0.78 μM, demonstrating that Digitoxin was an effective Na^+^/K^+^-ATPase inhibitor, which was consistent with previous studies [24,25].

### 2.2. Digitoxin Induced Cell Cycle Arrest in H1975

**Figure 2:**
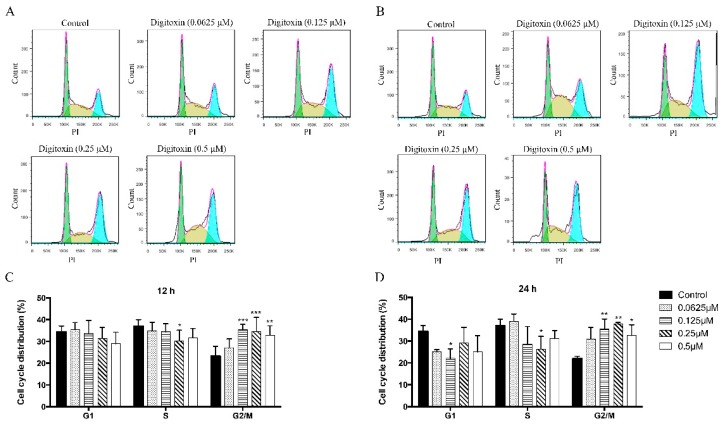
Digitoxin induced cell cycle arrest in H1975 cells. (**A**) H1975 cells were treated with Digitoxin at different concentrations (0, 0.0625, 0.125, 0.25, 0.5 μM) for 12 h. Cells were stained with PI and cell cycle arrest was detected by flow cytometry; (**B**) Cells were stained and collected for cell cycle arrest assay at 24 h after treatment with Digitoxin in the indicated concentrations; (**C**) Statistical analysis of cell cycle distribution in 12 h; (**D**) Statistical analysis of cell cycle distribution in 24 h. All data was presented as mean ± SEM (*n* = 3, * *p* < 0.05, ** *p* < 0.01, *** *p* < 0.001).

The mechanism of the anti-cancer effect of Digitoxin on H1975 has not yet been defined. To determine the mechanism of Digitoxin, we examined whether Digitoxin has a cell cycle arrest effect in H1975 cells. Cells were treated with the indicated concentrations of Digitoxin for 12 and 24 h, and stained with Propidium iodide (PI). The samples were analyzed by flow cytometry. The results showed that after 12 h treatment, the percentage of cells in G0/G1 phase slightly decreased while increased in G2/M phase (Figure 2A,C). Moreover, a more apparent trend was found after treatment for 24 h. Digitoxin-treated cells exhibited a 15% of increase in G2/M phase accompanied by a significant reduction in G0/G1 phase at high dose (Figure 2B,D). These results indicated that Digitoxin-induced inhibition mechanism is likely due to causing G2/M cell cycle arrest in H1975 cells in dose dependent manners.

### 2.3. Effects of Digitoxin on Cell Cycle Regulatory Proteins in H1975

**Figure 3:**
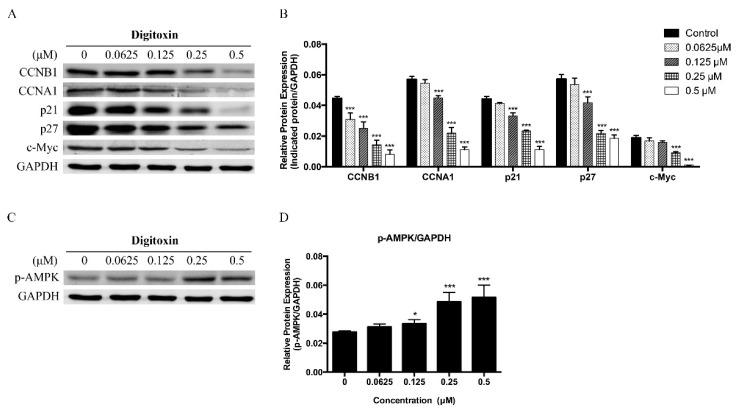
Digitoxin significantly regulated cell cycle-related proteins in H1975 cells. (**A**) H1975 cells were treated with Digitoxin at different concentrations (0, 0.0625, 0.125, 0.25, 0.5 μM) for 24 h. Protein levels of CCNB1, CCNA1, p21, p27, c-Myc and GAPDH by western blotting; (**C**) The protein of p-AMPK were determined by western blotting, and GAPDH was considered as a loading control; (**B**,**D**) Statistical analysis of CCNB1, CCNA1, p21, p27, c-Myc and p-AMPK. All data was presented as mean ± SEM (*n* = 3, * *p* < 0.05, *** *p* < 0.001). At least three independent experiments were performed.

To further clarify the underlying mechanism of Digitoxin in inducing cell cycle arrest in H1975, we examined the effect of Digitoxin on the expression of several cell cycle regulatory proteins. As shown in Figure 3A,B, Digitoxin significantly decreased the protein content of cyclin B1 (CCNB1) and cyclin A1 (CCNA1) resulting in G2/M phase arrest, which were consistent with the results of cell cycle arrest data detected by flow cytometry.

We also determined the effect of Digitoxin on modulating p21, p27 and phosphor-AMPK (p-AMPK) proteins. Western blotting results showed that Digitoxin remarkably down-regulated the expression of p21 and p27, both of which have been defined as cyclin-dependent kinase inhibitors (CKIs). It has been published that overexpression of p21 and p27 suppressed DNA synthesis, which arrested cell cycle in G1 phase and thus inhibited cell proliferation [26,27]. Therefore, the inhibition effect of Digitoxin was not caused by induction of G1-arrest. As the key regulator of cellular metabolism and energy homeostasis, AMPK was also associated with cell cycle and cell proliferation. Our results showed that Digitoxin was found to activate p-AMPK in a dose-dependent manner (Figure 3C,D). Altogether, our data demonstrated that Digitoxin affected the cell cycle arrest in G2/M phase by altering the expression of CCNB1 and CCNA1 and may be also associated with AMPK activation.

To verify the inhibition of cell growth by Digitoxin in H1975, we performed western blotting to examine the expression level of c-Myc. It has been reported that c-Myc plays a major role in Myc family to target DNA transactivation and promote cell proliferation [28]. As shown in Figure 3A, by 24 h, c-Myc expression was significant reduced by Digitoxin. Loss of c-Myc suggested that Digitoxin has suppressive effect on the proliferation of H1975 cells.

### 2.4. Digitoxin Inhibited Microtubule Formation

**Figure 4:**
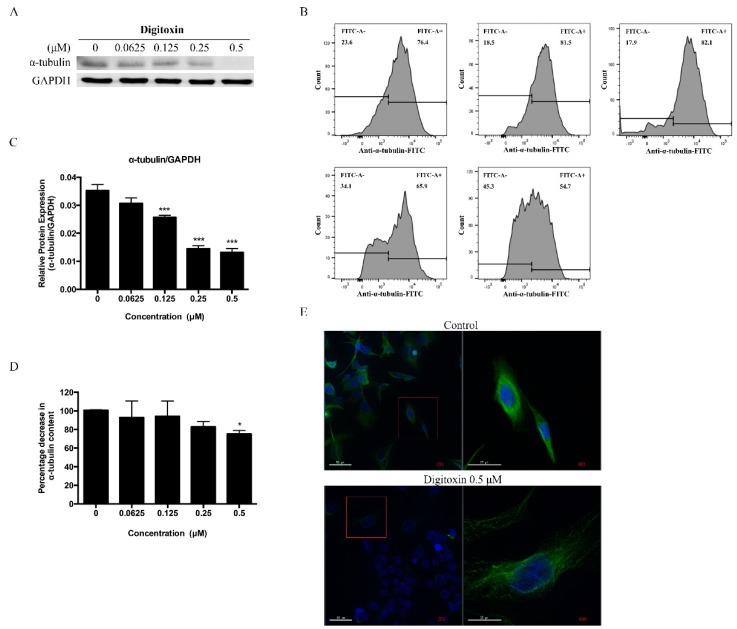
Digitoxin suppressed tubulin polymerization in H1975 cells. (**A**) H1975 cells were treated with Digitoxin at different concentrations (0, 0.0625, 0.125, 0.25, 0.5 μM) for 24 h. Western blotting was used to detect the protein content of α-tubulin; (**B**) H1975 cells were treated with Digitoxin for different concentration as indicated, and then cells were incubated with primary α-tubulin antibodies and stained with anti-mouse-FITC secondary antibodies. The fluorescence intensity was analyzed by flow cytometry; (**C**) Statistical analysis of α-tubulin; (**D**) Statistical analysis of the relative decrease in α-tubulin intensity; (**E**) Cells were treated with or without Digitoxin for 24 h and processed with immunofluorescence staining. Images were captured by Delta Vision Live Cell Imaging System (20×, 60× objective magnification). All data was presented as mean ± SEM (*n* = 3, * *p* < 0.05, *** *p* < 0.001).

Microtubule is known as a key regulator of cell cycle progression. Therefore, we determined the effect of Digitoxin on α-tubulin protein content and intensity. As measured by western blotting and flow cytometer, α-tubulin was remarkably reduced (Figure 4A,B). Furthermore, the intensity of α-tubulin was visualized by immunofluorescence. As shown in Figure 4E, the control group displayed a normal microtubule network that fully extended to the whole cell, while Digitoxin-treated cells weaken the microtubules incorporation and showed a severely disruption and disorganized structure. Collectively, these results suggested that Digitoxin might target on α-tubulin, suppress microtubule formation, prevent mitotic spindle to break chromosome and consequently interfere with mitosis.

### 2.5. Effect of Digitoxin on Cell Proliferation

**Figure 5:**
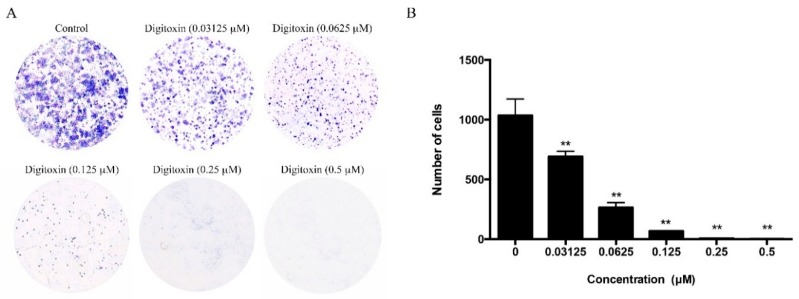
Digitoxin inhibited cell proliferation. (**A**) Data were shown as representative photomicrographs of colony formation assay after treatment with Digitoxin at different concentration (0.03125, 0.0625, 0.125, 0.25, 0.5 μM); (**B**) Statistical analysis of colony formation assay. Statistical analysis was conducted using one-way analysis of variance (ANOVA). All data was presented as mean ± SEM (*n* = 3, ** *p* < 0.01).

Since Digitoxin is able to modulate cell cycle arrest, we try to determine whether it regulates cell proliferation. We used colony formation assay to detect the role of Digitoxin on clonogenic survival. As shown in Figure 5, Digitoxin treatment significantly attenuated cells in a dose-dependent manner. When cells were treated with 0.5 μM of Digitoxin, the percentage of cell number remarkably decreased by 95% approximately compared with control cells, suggesting that Digitoxin reduced cell proliferation and colony growth.

## 3. Discussion

TCM has been widely used in treatment of complex diseases, including diabetes, neurodegenerative disease and cancer [29,30,31]. As such, it could be used as important resources for new drug discovery [32].

Digitalis containing cardiac glycosides has been used for the treatment of heart diseases for over 200 years [33]. An ancient Chinese remedy that employs an extract of Bufo toad secretions contains several cardiac glycosides and is still being used today for managing cancerous conditions [34,35,36]. Additionally, in 1980s, digitalis was firstly found to have anti-cancer effect in breast cancer [37]. Since then, more and more researchers put their efforts on investigating the anti-cancer mechanism of digitalis including Ouabain, Digoxin, Digitoxin and so on. For instance, it has been reported that ouabain inhibited cell proliferation and induced cell death by activation of c-Jun *N*-terminal kinase (JNK) pathway [38]. Digitoxin was described as a target of Na^+^/K^+^-ATPase pump inhibitor in the last century. In addition, its cytotoxicity was reported colsely related inhibition effect on Na^+^/K^+^-ATPase [24,25]. It indicated that Na^+^/K^+^-ATPase not only signals the ion pump, but also participates as a signaling transducer in several downstream pathways, including EGFR, mitogen-activated protein kinases (MAPK), and phosphoinositide 3-kinase (PI3K) pathways [39]. It has been investigated that digitalis-induced activation of EGFR-MAPK pathway by binding with Na^+^/K^+^-ATPase. It led to cell cycle arrest by resulting in up-regulation of p21, also known as CKI1 [40]. In addition, digoxin has been reported for suppressing cancer through inhibiting multiple proto-oncogene tyrosine-protein kinase (Src)-related signaling pathways in NSCLC cell lines [41]. Digitoxin, a member of digitalis family, showed that it can increase level of intracellular calcium by inhibiting the sodium-potassium adenosine triphosphatase (Na^+^/K^+^-ATPase) complex in myocardial cells [42]. It has been reported that a low dose of Digitoxin resulted in activation of cell apoptosis pathway in H460 cells, but no less sensitive on primary human lung epithelial cells and non-tumorigenic human lung epithelial cells [43]. Nevertheless, the mechanism of it in the treatment of cancer has not been fully defined yet.

Although plenty of reports about the anti-cancer function of digitalis have been published, few articles about EGFR^T790M/L858R^ were on digoxin [41] and ouabain [38]. In NSCLC, many studies were mostly focused on A549 [38,41,44,45,46,47,48,49,50,51,52] and H460 cells with WT EGFR [41,43,53,54]. To our knowledge, no research report has addressed the effect of Digitoxin on Gefitinib resistant EGFR^T790M/L858R^ mutated H1975 cells. In our study, we have applied cell-based method to large-scale screening of a TCM compound library consisting of 800 compounds and used NSCLC H1975 and A549 cell lines as screening tools, we have shortlisted 24 candidates with CC_50_ value less than 2.5 μM, one of the compounds, Digitoxin, was found effective in Gefitinib resistance H1975 with EGFR^T790M/L858R^ double mutation.

By using quantitative flow cytometry analysis, we found that Digitoxin remarkably increased the percentage of cells at G2/M phase coherent with a decrease at G1 phase. Moreover, these results indicated that Digitoxin arrested cell cycle at G2/M phase consequently inhibited mitosis. As a promising target, regulation of cell cycle checkpoints significantly contributed to the treatment of cancer [55]. In eukaryotic cells, cell cycle evolved an integrated protective mechanism, which regulated by a group of cyclin, cyclin-dependent kinases (CDK), and CKIs [56,57,58]. Besides that, the cell cycle checkpoint is a cell survival monitoring system, which inspects DNA damage and defective spindle formation, controlling gene expression and maintaining genomic stability [59,60]. Whether cells can pass the G2/M checkpoint depends on the level of CCNB1, which is the initial activator and pivotal regulator, leading to regulation and activation of CDK1/CCNB1 complexes. Phosphorylation of these complexes further enforced cell cycle through the checkpoint and facilitate transition from G2 phase into mitosis [61,62]. Therefore, restraining the expression of CCNB1 arrested cell cycle at G2/M phase prevents cell cycle progression. Yuan J and others performed the experiments to reduce CCNB1 expression by RNA interference, which caused inhibition of cell proliferation by arresting cells at G2 phase, suggesting that the expression level of CCNB1 played an important role in initial regulation of mitosis [62]. Besides, different from CCNB1, CCNA1 functions in S and G2/M phases, which is due to its two different phosphorylated substrates, CDK1 and CDK2, subsequently conducing tumor cells proliferation [63]. Our data demonstrated that Digitoxin arrested the H1975 cell cycle. We further observed that Digitoxin significantly down-regulated CCNB1 and CCNA1, which could explain our flow cytometry results of G2 phase cell cycle arrest. By western blotting, we also found that both p21 and p27 were suppressed by Digitoxin.

In addition, activation of AMPK provides an inhibition effect on CDK, p21 and p27 [64]. It has been reported that AMPK effected ataxia telangiectasia mutated (ATM) signals to mediate p53, p21 and p27, consequently targeted to cell cycle checkpoints [65,66,67,68]. The activity of AMPK also varies at different stages of the cell cycle, it progressively increases from G1 phase to G2/M phase, reaching the peak at G2/M, and then decreases when cells entering G1/S stage [64,69]. Our study observed that Digitoxin induced cell cycle arrest at G2/M phase, which could be a combined mechanistic effect of cell cycle regulators and AMPK activation to maintain cell cycle at G2/M. Furthermore, overexpression of AMPK is also associated with a lack of mammalian target of rapamycin (mTOR) level, resulting in suppression of cell growth, proliferation and protein synthesis [66,70,71]. Thus, it may explain the colony formation suppression effect by Digitoxin.

Moreover, AMPK is also defined as a member of microtubule affinity-regulating kinase (MARK)/PAR kinase subfamily, leading to phosphorylation of microtubule-associated proteins (MAPs), which controls microtubule dynamics [72]. The microtubule is a key component of the cytoskeleton, formed by the polymerization of a dimer of two globular proteins, alpha and beta tubulin [73]. As structural elements, microtubules support cell stabilization and function as mitotic spindle in eukaryotic cells to segregate their chromosomes during cell division [74,75,76,77]. By α-tubulin tyrosination and acetylation, microtubules maintain a dynamic balance [78,79,80,81], loss of microtubule also leads to cell cycle arrest. Our studies showed that Digitoxin-induced G2 phase arrest is associated with AMPK activation and loss of α-tubulin and microtubles.

Despite the fact that Digitoxin had strong cytotoxic effect on H1975, it showed no selectively between A549. Similar to our findings, it has been published that two derivatives of the digitalis, digoxin and ouabain, also induced moderate G2/M arrest in A549 and H460, by activation of AMPK-mTOR pathway [50]. It seems that digitalis families could influence the cell cycle both in the presence or absence of the EGFR mutant. Nevertheless, since cell mitotic, migration and growth strongly relied on microtubule, nowadays, anti-microtubule polymerization is emerging as a new anti-cancer therapy. For example, a novel microtubule-targeting agent, 7-deazahypoxanthines, showed statistically significant tumor size reduction in a colon cancer mouse model [82]. Denning *et al.* also refereed anti-tubulin drugs as life-saving chemotherapeutics that can kill cancer cells by stabilizing or disrupting microtubules [83]. Our novel discovery of Digitoxin as a promising anti-tubulin formation agent on Gefitinib resistance NSCLC has opened new anti-genfitnib resistance intervention option.

## 4. Materials and Methods

### 4.1. Materials

The natural product compound library consisting of 800 single purified compounds were purchased from MicroSource Discovery Systems/Topscience (Shanghai, China). Digitoxin powder was purchased from Sigma Aldrich (St. Louis, MI, USA). MTT powder and Dimethyl sulfoxide (DMSO) was purchased from Acros Organics (Morris Plains, NJ, USA). Adenosine 5′-Triphosphatase was purchased from Sigma Aldrich. Radioimmunoprecipitation (RIPA) lysis buffer (10×) and the primary antibodies of CCNB1, CCNA1, p21, p27, c-Myc and GAPDH were purchased from Cell Signaling Technology (Danvers, MA, USA). The primary antibodies of phospho-AMPK, α-tubulin were purchased from Santa Cruz (Dallas, TX, USA). The secondary antibodies of anti-rabbit and anti-mouse were purchased from Odyssey (Belfast, ME, USA). Fluorescein-conjugated goat anti-rabbit and mouse anti-bodies were purchased from Odyssey (Belfast, ME, USA) and Invitrogen (Waltham, MA, USA). Propidium iodide (PI) staining kit was purchased from BD Biosciences (San Jose, CA, USA). RNase A was purchased from Sigma Aldrich.Ten percent fetal bovine serum (FBS), 100 U/mL penicillin and 100 μg/mL streptomycin were purchased from Gibco (Oklahoma, ME, USA). A complete mini, EDTA-free tablet was from Roche (Mannheim, Germany). DCTM protein assay kit was purchased from Bio-Rad (Hercules, CA, USA). Nitrocellulose (NC) membrane was purchased from GE Healthcare (Waukesha, WI, USA). Crystal violet was purchased from Amresco (Solon, OH, USA).

### 4.2. Cell Culture

H1975, A549 NSCLC cell lines, and CCD-19Lu the normal lung fibroblast cells of lung, all of these cell lines were purchased from ATCC. H1975 and A549 cell lines were cultivated with RPMI 1640 medium supplemented with 10% FBS, 100 U/mL penicillin and 100 μg/mL streptomycin. The CCD-19Lu cells were cultivated with MEM medium supplemented with 10% FBS, 100 U/mL penicillin and 100 μg/mL streptomycin. All cells were cultivated at 37 °C in a humidified atmosphere of 5% CO_2_.

### 4.3. MTT Assay

Cells were seeded with 3 × 10^3^ cells/well in a 96-well plate and allowed to adhere after overnight incubation. Cells were treated with various concentrations of compounds (0, 0.625, 1.25, 2.5, 5 μM) and with vehicle control, DMSO, for 72 h. Ten μL of MTT solution were added to each well and incubated at 37 °C for 4 h. Then, 100 μL of resolved solution (10% SDS and 0.1 mM HCL) was added to each well and incubated at 37 °C for 4 h to solubilize the formazan crystals. Absorbance of plates was measured at 570 nm (absorbance) and 650 nm (reference) with Tecan microplate reader (Morrisville, NC, USA). Cell viability (%) was calculated by percentage of the absorbance of the treated group divided by control group. It was expressed as = [OD of treated group/OD of control group] × 100%, and the viability of control group was considered as 100%.

### 4.4. Na^+^/K^+^ ATPase Enzymatic Activity Assay

The enzymatic activity of Na^+^/K^+^ ATPase was measured as the rate of release of inorganic phosphate during ATP hydrolysis. Indicated concentrations (0, 0.25, 0.5, 1 μM) of Digitoxin were mixed with 67.5 μL Tris HCl Buffer, which contains Ethylenediaminetetraacetic Acid and Magnesium Chloride and made as testing buffer. Ten μL Na^+^/K^+^ ATPase and 2.5 μL KCl/NaCl were gently mixed with the testing buffer and incubated for 30 min at 37 °C. After that, 5 μL ATP was added and the reaction was started by incubated again for 15 min at 37 °C. After the final incubating, spin the mixture carefully, followed by centrifugation at 1000 rpm for 3 min. Transferred 50 μL supernatant of each sample to a 96-well plate with 100 μL Taussky-Shorr Reagent to terminate reaction. Absorbance of plates was measured at 660 nm with Tecan microplate reader.

### 4.5. Cell Cycle Analysis

H1975 cells were plated with 2.0 × 10^5^ cells/well at a 6-well plate and cultured overnight for attachment. Cells were treated with Digitoxin at 0, 0.0625, 0.125, 0.25, 0.5 μM for 12 h and 24 h respectively. After treatment, all cells were harvested by trypsinization, and collected by centrifugation. After removing all suspension, cells were washed by PBS. Cells pellets were re-suspended in 70% ethanol at 4 °C for 30 min. Cells were centrifuged at 1000 rpm for 5 min to remove all the ethanol. Each cell pellet was re-suspended in 500 μL PI staining solution at 37 °C for 30 min in dark. Then, cells were washed in PBS twice. Cells were re-suspended in 300 μL 1 × binding buffer and transferred to the flow cytometer (BD FACS Aria III).

### 4.6. Western Blot Analysis

H1975 cells were plated with 2.0 × 10^5^ cells/well at a 6-well plate and cultured overnight for attachment. Cells were lysed in 1 × RIPA lysis buffer with proteinase inhibitor and phosphatase inhibitors added, and were scraped off from the plate by a plastic cell scraper. All the lysate was mixed well and kept in ice then transferred to a new tube. The lysate was centrifuged at 4 °C 12,000 rpm for 5 min. After centrifugation, the suspension with protein lysate was kept on ice. Protein concentration was quantitatively measured by DCTM protein assay kit, the supernatant was transferred into a new tube and mixed with 5 × loading buffer. Each sample was boiled at 100 °C for 5 min. Twenty five μg of each protein samples were loaded into the well of a 10% SDS-PAGE gel with one lane of 3 μL protein molecular weight marker. The gel was run for 20 min at 80 V for stacking, and then added to 120 V for protein separation. After separation, the proteins from the gel were transferred to a NC membrane for 2 h at 300 mA, the membrane was blocked with 5% non-fat milk diluted with 1 × TBST (0.1% Tween 20 in Tris-buffered saline) at room temperature for 1 h and washed with 1 × TBST for three times. Membranes were incubated with primary antibodies at 1:1000 dilution at 4 °C overnight. After washing the membrane 3 times with 1 × TBST, the membranes were incubated with secondary antibodies at 1:10,000 dilutions for 1 h at room temperature. GAPDH was used as endogenous loading control for normalization. The signal intensity of the membranes was detected by LI-COR Odyssey scanner (Belfast, ME, USA).

### 4.7. Immunofluorescence Flow Cytometry

To identify the quantity of α-tubulin caused by Digitoxin, H1975 cells were cultured in 6-well plate, each well of 1.5 × 10^5^ cells. After exposure to Digitoxin of 24 h. the cells were collected by centrifugation and washed once with PBS. After being fixed with 4% paraformaldehyde in PBS, the cells were permeabilized by ice-cold 100% methanol for 30 min on ice. Centrifuged after having been washed by incubation buffer (0.5 g Bovine Serum Albumin (BSA) in 100 mL 1 × PBS). α-tubulin was stained with α-tubulin antibody overnight at 4 °C. Washed by centrifugation in 2–3 mL incubation buffer. Resuspend cells in fluorescein isothiocyanate (FITC)-conjugated secondary antibody, diluted in incubation buffer at 1:250 dilution. Incubate for 1 h at room temperature in dark. Cells were washed by centrifugation in 2–3 mL incubation buffer. Resuspend cells in PBS and measured by flow cytometer.

### 4.8. Indirect Immunofluorescence Microscopy

H1975 cells were grown on glass coverslips in 6-well plate, each well of 1.5 × 10^5^ cells. After exposure to Digitoxin, cells were washed once with PBS and then fixed with 4% paraformaldehyde in PBS for 15 min. The fixed cells were washed twice with PBS and then permeabilized with methanol for 2 min. Then rehydrated with PBS and incubated with α-tubulin (1:500) primary antibody in 5% bovine serum albumin at 4 °C overnight. Excess antibodies were removed by multiple washings with PBS. Added conjugated anti-mouse IgG (1:500) antibody as secondary antibody. After incubation for 2 h at room temperature, cells were stained by hoechst (1:10,000) for 10 min. Remove the hoechst washed with PBS twice, coverslips were mounted onto microscope slides with fluor save regent. Images were captured with Delta Vision Live Cell Imaging System by 20×, 60× objective respectively.

### 4.9. Colony Formation Assay

H1975 cells were seeded at a density of 5 × 10^3^ cells/well in a 6-well plate, and cultured overnight for attachment. Exposed on Digitoxin with concentration (0, 0.03125, 0.0625, 0.125, 0.25, 0.5 μM) for 7 days. Then it was changed medium at every 72 h. At the 10th day, the colonies were washed by PBS once, and fixed with 4% PFA. The colonies were washed by PBS twice, stained with crystal violet solution for 20 min, and washed. The number of colonies formed were counted in each group.

### 4.10. Statistical Analysis

All data represent mean values of at least three independent experiments and were expressed as mean ± SEM. The statistical significant differences were analyzed by one-way ANOVA followed by Bonferroni for comparison tests, using Graph Prism Version 6.0 software (GraphPad Software, Inc., San Diego, CA, USA). * *p* < 0.05, ** *p* < 0.01, *** *p* < 0.001 were considered as significant.

## 5. Conclusions

In conclusion, by using drug screening, *in vitro* functional assays and mechanism studies, we discovered that Digitoxin can lead to G2 phase arrest in H1975, a Gefitinib-resistant NSCLC cells with EGFR double mutations. We innovatively found that Digitoxin could directly suppress microtubule formation by reducing α-tubulin. Finally, we considered that Digitoxin has potential as the anti-cancer therapy with multifunction.

## Figures and Tables

**Table 1:** CC_50_ values of twenty-four shortlisted candidate compounds in H1975 and A549 cell lines.

CC_50_ Value (μM)		
Candidate Compounds	H1975 (EGFR L858R+T790M)	A549 (EGFR WT)
Digitoxin	0.19 ± 0.06	0.18 ± 0.02
Daunorubicin	0.24 ± 0.11	0.23 ± 0.03
Proscillaridin	0.32 ± 0.21	0.042 ± 0.02
Pyrromycin	0.33 ± 0.13	0.031 ± 0.003
Strophanthidinic acid lactone acetate	0.36 ± 0.17	0.08 ± 0.03
Plumbagin	0.39 ± 0.13	2.52 ± 1.84
Camptothecin	0.41 ± 0.17	0.11 ± 0.02
Digydrocelastryl Diacetate	0.52 ± 0.14	2.16 ± 1.16
Celastrol	0.53 ± 0.15	1.49 ± 0.79
Valinomycin	0.64 ± 0.26	0.43 ± 0.20
Antimucin	0.64 ± 0.38	4.23 ± 2.30
Picropodophyllin	0.75 ± 0.33	1.02 ± 0.54
Anthothecol	0.79 ± 0.22	2.46 ± 1.31
5alpha-cholestan-3beta-ol-6-one	0.81 ± 0.39	1.02 ± 0.58
Patulin	1.03 ± 0.29	1.62 ± 0.44
Rotenone	1.06 ± 0.46	0.82 ± 0.36
Gambofic acid	1.15 ± 0.69	3.85 ± 1.81
Strophanthidin	1.19 ± 0.43	0.16 ± 0.03
Estrone Benzoate	1.40 ± 0.39	0.40 ± 0.14
Picropodophyllin Acentate	1.57 ± 0.69	0.67 ± 0.25
Paclitaxel	1.67 ± 0.80	0.79 ± 0.49
Monensin Sodium	1.71 ± 0.76	1.16 ± 0.35
Dihydrorotenone	2.27 ± 1.10	2.21 ± 1.36
Isorotenone	2.49 ± 1.2	1.35 ± 0.796
